# Mitigating Inhomogeneity and Tailoring the Microstructure of Selective Laser Melted Titanium Orthorhombic Alloy by Heat Treatment, Hot Isostatic Pressing, and Multiple Laser Exposures

**DOI:** 10.3390/ma14174946

**Published:** 2021-08-30

**Authors:** Igor Polozov, Kirill Starikov, Anatoly Popovich, Vadim Sufiiarov

**Affiliations:** Institute of Mechanical Engineering, Materials, and Transport, Peter the Great St. Petersburg Polytechnic University, Polytechnicheskaya 29, 195251 St. Petersburg, Russia; kirill.starikov@gmail.com (K.S.); director@immet.spbstu.ru (A.P.); sufiyarov_vsh@spbstu.ru (V.S.)

**Keywords:** intermetallic alloy, additive manufacturing, Ti-22Al-25Nb, platform preheating, powder bed fusion, heat treatment

## Abstract

Titanium orthorhombic alloys based on intermetallic Ti_2_AlNb-phase are attractive materials for lightweight high-temperature applications. However, conventional manufacturing of Ti_2_AlNb-based alloys is costly and labor-consuming. Additive Manufacturing is an attractive way of producing parts from Ti_2_AlNb-based alloys. High-temperature substrate preheating during Selective Laser Melting is required to obtain crack-free intermetallic alloys. Due to the nature of substrate preheating, the temperature profile along the build height might be uneven leading to inhomogeneous microstructure and defects. The microstructural homogeneity of the alloy along the build direction was evaluated. The feasibility of mitigating the microstructural inhomogeneity was investigated by fabricating Ti_2_AlNb-alloy samples with graded microstructure and subjecting them to annealing. Hot isostatic pressing allowed us to achieve a homogeneous microstructure, eliminate residual micro defects, and improve mechanical properties with tensile strength reaching 1027 MPa and 860 MPa at room temperature and 650 °C, correspondingly. Annealing of the microstructurally graded alloy at 1050 °C allowed us to obtain a homogeneous B2 + O microstructure with a uniform microhardness distribution. The results of the study showed that the microstructural inhomogeneity of the titanium orthorhombic alloy obtained by SLM can be mitigated by annealing or hot isostatic pressing. Additionally, it was shown that by applying multiple-laser exposure for processing each layer it is possible to locally tailor the phase volume and morphology and achieve microstructure and properties similar to the Ti2AlNb-alloy obtained at higher preheating temperatures.

## 1. Introduction

Orthorhombic titanium alloys based on the intermetallic O-phase (Ti_2_AlNb) are considered promising materials to replace nickel-based alloys due to their high specific strength, creep resistance, and oxidation resistance with the maximum operating temperatures of 650–700 °C [1,2]. Ti-22Al-25Nb (at. %) is one of the most-investigated Ti_2_AlNb-based alloys, but alloying with Mo, Zr, W, and Si is needed to improve its oxidation resistance and creep strength [3,4,5].

Poor machinability of these alloys, a tendency toward segregation, and limitations of conventional manufacturing techniques in terms of final product geometry make Additive Manufacturing (AM) processes, particularly Selective Laser Melting (SLM), an attractive method for the production of complex intermetallic parts. Conventional welding methods have been used to fuse intermetallic titanium alloys by laser or electron-beam melting; however, the process remains challenging due to the susceptibility of these alloys to cold cracking [6].

AM of orthorhombic titanium alloys has been limited so far due to the challenges in terms of cracking susceptibility [7], microstructural homogeneity [8], and feedstock powder availability [9]. Elemental powder blends were used in the SLM process without high-temperature platform preheating to fabricate a Ti-22Al-25Nb alloy via in situ synthesis [10,11]. While this approach allowed for the obtainment of a Ti_2_AlNb-based alloy, the mechanical properties were poor due to cracking of the alloy. Due to high thermal stresses occurring during the SLM process, it is necessary to use high-temperature platform preheating to suppress cracking and obtain defect-free intermetallic alloys [7,12]. As shown in the previous research [7], 200 °C platform preheating temperature during the SLM of an orthorhombic alloy was not sufficient to mitigate cracking and the preheating temperature should be maintained above 600 °C to avoid cold cracking. At the same time, the microstructure and properties of the alloy significantly depend on the preheating temperature and SLM process parameters [7]. In addition, as the height of the manufactured part increases, the temperature distribution may not be uniform across the height of the part, since high-temperature preheating is realized by preheating the platform during the SLM process [13,14] as opposed to electron-beam melting (EBM) where each layer of the powder bed is preheated by an electron beam [15]. This, in turn, leads to inhomogeneous microstructure, the presence of micro defects, and reduced mechanical properties [10,16]. As shown in [8], Ti-22Al-25Nb alloy fabricated by laser AM technology without platform preheating exhibited inhomogeneous microstructure and properties along the building direction due to the thermal history of the process. Additionally, it may be necessary to carry out post-treatment in the form of hot isostatic pressing and heat treatment to improve the mechanical characteristics of orthorhombic titanium alloys obtained by AM processes and the post-treatment parameters can significantly influence the microstructure and phase composition of the alloy, leading to B2, B2 + O, and B2 + O + α_2_ microstructures with equiaxed grains and acicular, lamellar or Widmanstatten morphology [17,18].

The issue of microstructural homogeneity of orthorhombic titanium alloys along the building direction obtained by SLM with high-temperature platform preheating has not been investigated so far. For further applications of high-temperature platform preheating during the SLM process, it is necessary to understand if the microstructure of the alloy is homogeneous along the building direction and if the microstructural inhomogeneity can be mitigated by post-processing.

In this work, high-temperature platform preheating was used to fabricate the titanium orthorhombic alloy by SLM with the building direction parallel to the tensile direction. The microstructural homogeneity of the alloy along the build direction was evaluated. The feasibility of mitigating the microstructural inhomogeneity was investigated by fabricating Ti_2_AlNb-based alloy samples with graded microstructure and subjecting them to annealing. In this case, the graded samples were produced by using different platform preheating temperatures for different areas of the samples.

Additionally, laser-scanning strategies with multiple scans for each layer were used to locally tailor the microstructure of the alloy by promoting in situ heat treatment during the SLM process. It was established that applying a scanning strategy with multiple-laser exposure can be used to tailor morphology and volume fraction of the Ti_2_AlNb-based alloy and to obtain the alloy with microstructure and properties similar to the alloy produced with a higher-platform preheating temperature.

## 2. Experimental Procedures

Spherical powder of Ti-24Al-25Nb-1Zr-1.4V-0.6Mo-0.3Si (at. %) orthorhombic alloy produced by electrode induction gas atomization and supplied by AMC Powders (Beijing, China) was used as the feedstock material in the SLM process. The oxygen content of the powder was 0.14 wt. % as measured by the inert-gas fusion infrared method with a LECO TC-500 analyzer (LECO, Saint Joseph, MI, USA). The particle size of the powder ranged from 14 to 52 µm with a mean particle size d_50_ = 29 µm.

**Figure 1:**
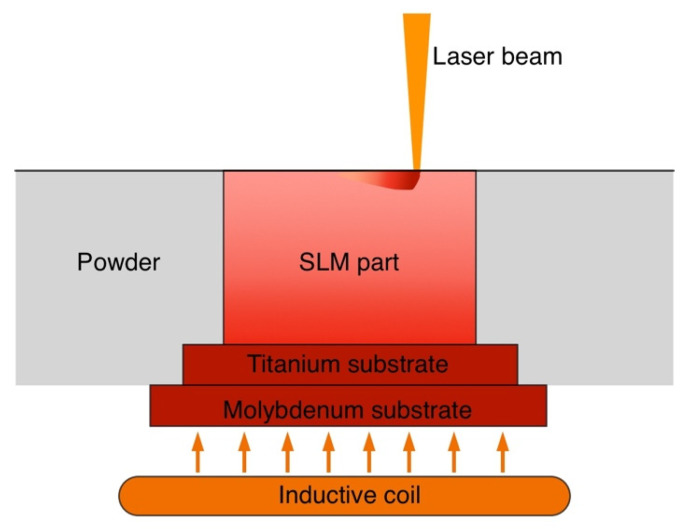
Schematic representation of the SLM process with a high-temperature inductive substrate preheating.

The SLM process was carried out using AconityMIDI (Aconity3D GmbH, Herzogenrath, Germany) SLM system equipped with a 1070 nm wavelength fiber laser with a maximum power of 1000 W. The samples were fabricated on a Ti-6Al-4V substrate, which was put on a molybdenum platform. The molybdenum platform was inductively preheated to a set temperature, which was continuously controlled by a thermocouple under the molybdenum platform. The titanium substrate was then conductively heated to the set temperature before starting the SLM process. The process is schematically shown in Figure 1. The process chamber was continuously flooded with high purity argon gas to achieve oxygen content in the chamber below 20 ppm. After the build process was finished, the platform and the samples were cooled to room temperature with a cooling rate of approximately 5 °C/min.

**Figure 2:**
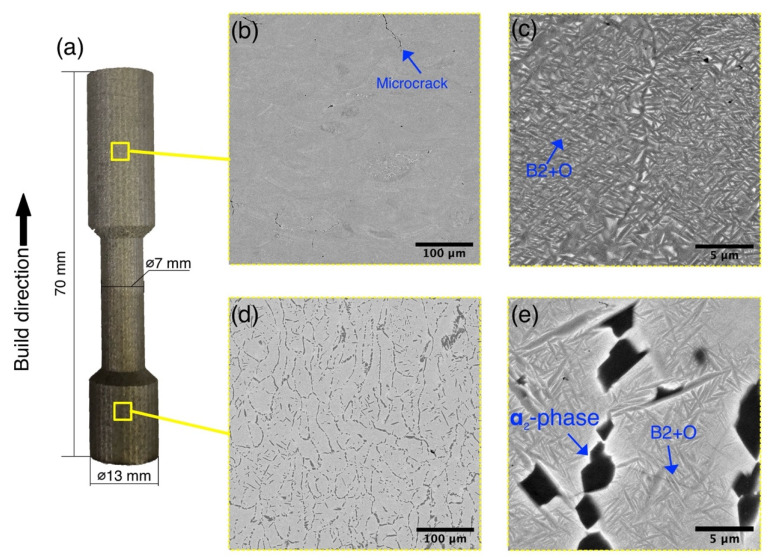
(**a**) A photograph of a tensile specimen built by SLM at 950 °C platform preheating and BSE-SEM images showing microstructures of (**b**,**c**) the top and (**d**,**e**) bottom parts.

Dog bone samples with a height of 70 mm were fabricated for tensile tests with a load direction oriented parallel to the build direction. The dimensions of the specimens are shown in Figure 2a. Before testing, the dog bone tensile samples were machined to achieve 30 mm gauge length and 6 mm diameter. Cylindrical samples with 15 mm diameter and 30 mm height were fabricated for microstructural evaluation. The following SLM process parameters were chosen based on the previous study [7]: 140 W laser power, 850 mm/s scanning speed, 120 µm hatch distance, 30 µm layer thickness, and 950 °C platform preheating temperature.

Additionally, in order to evaluate microstructural inhomogeneity of the material along the build height and effects of heat treatment on microstructure and properties of the material with inhomogeneous microstructure, microstructurally graded samples were fabricated using different platform preheating temperatures during the SLM process. Initially, the first 15 mm of the sample were produced at 950 °C preheating temperature. Then, the SLM process was paused, the platform was cooled off to 700 °C, and the remaining 15 mm were built.

In order to investigate the feasibility of the scanning strategy with multiple laser exposures to tailor the microstructure of the orthorhombic alloy during the SLM process via in situ heat treatment, samples were scanned for 10 additional times for each layer or every third layer were produced using a 700 °C platform preheating temperature. The number of additional scans was chosen based on the previous study using a TiAl-based alloy [19] where 10 additional scans allowed for the promotion of in situ heat treatment and microstructure modification. Scanning speed and hatch distance for additional scans were increased compared with the main scan in order to reduce the energy input and increase productivity. Additionally, a scanning strategy with single-laser exposure and higher preheating temperatures was used to produce the samples for comparison. Table 1 shows more detailed process parameters used for samples fabrication.

The microstructure of the fabricated samples was characterized using a Mira 3 LMU (TESCAN, Brno, Czech Republic) scanning electron microscope (SEM) in backscattered electrons (BSE) mode for a polished section of the sample prepared using a standard metallographic technique.

The phase composition of the powders and the fabricated samples was analyzed with a Bruker D8 Advance X-ray diffraction (XRD) meter (Bruker, Billerica, MA, USA) using Cu-Kα (λ = 1.5418 Å) irradiation.

The microhardness of the samples was measured using a Buehler VH1150 testing machine with a Vickers indenter at 500 g load and 10 s dwell time. At least five measurements at a randomized position for each sample were taken.

Room and high-temperature tensile tests were carried out using a universal testing machine (Zwick/Roell Z100, Ulm, Germany) with a tensile strain of 0.3 mm/min. The tensile direction was perpendicular to the build direction. Three tensile specimens per point were used to evaluate average values.

Hot isostatic pressing (HIP) of the samples was carried out at 1050 °C, 160 MPa pressure for 3 h followed by furnace cooling.

Heat treatment of the microstructurally graded samples was carried out using a vacuum furnace at 1050 °C for 1.5 h followed by furnace cooling.

## 3. Results and Discussion

### 3.1. Orthorhombic Alloy Produced with Single Laser Scanning

#### 3.1.1. Microstructural Inhomogeneity of the Alloy Obtained with a High-Temperature Platform Preheating

Using the high-temperature platform preheating during the SLM process, vertical dog bone specimens were produced from the Ti_2_AlNb-alloy powder with a tensile direction parallel to the build direction as shown in Figure 2a. During the SLM process, the titanium platform preheating temperature was maintained at 950 °C. The produced specimens were then used for tensile tests as well as microstructural investigation of bottom and top areas.

As can be seen from Figure 2, the microstructure of the alloy significantly differed for the bottom and top areas of the sample. The bottom area, which is closer to the preheated platform, featured a B2 + O + α_2_ microstructure with relatively coarse prior B2 grains as can be seen in Figure 2d (gray contrast). Under higher magnifications, a fine acicular B2 + O microstructure can be seen (Figure 2e). Coarse α_2_ (Ti_3_Al) phase precipitates appeared along the prior B2 grain boundaries, suggesting that during the SLM process aging of the alloy occurred. Precipitation of the α_2_ phase as a result of B2 phase decomposition in orthorhombic titanium alloys usually requires relatively long holding times in the B2 + O + α_2_ or B2 + α_2_ phase region due to slow diffusivity of Nb in the alloy [20]. The SLM fabrication process of the specimens took approximately 12–15 h, which coupled with the high-temperature platform preheating and laser-induced heating cycles and promoted the decomposition of the B2 phase and the precipitation of α_2_ phase in the bottom area.

As can be seen from Figure 2b,c, the top area of the fabricated specimen featured different microstructure morphology compared with the bottom area. A fine lamellar B2 + O microstructure can be seen at high magnifications (Figure 2c). It can be seen that the top area consisted of a significantly higher amount of O (Ti_2_AlNb)-phase precipitates (dark grey contrast) compared with the bottom area. As shown in several publications, the volume fraction and size of O-phase precipitates significantly depend on the processing temperature and/or heat-treatment conditions [20]. A lower O-phase volume fraction corresponds to higher annealing temperatures in the case of Ti_2_AlNb-based alloys [21]. This suggests that the top area of the specimen was processed at lower temperatures closer to the single O-phase region during the SLM build compared with the bottom area. This resulted in a nonhomogeneous microstructure of the alloy along the build direction. Moreover, some microcracks were found in the top area of the sample, suggesting that the preheating temperature at these layers was lower compared with the bottom part. Thus, the SLM process of the titanium intermetallic alloy with a high-temperature substrate preheating resulted in a non-homogeneous microstructure due to uneven temperature distribution along the build height.

#### 3.1.2. Effect of Heat Treatment on Microstructure and Microhardness of the Orthorhombic Alloy with a Graded Microstructure

**Figure 3:**
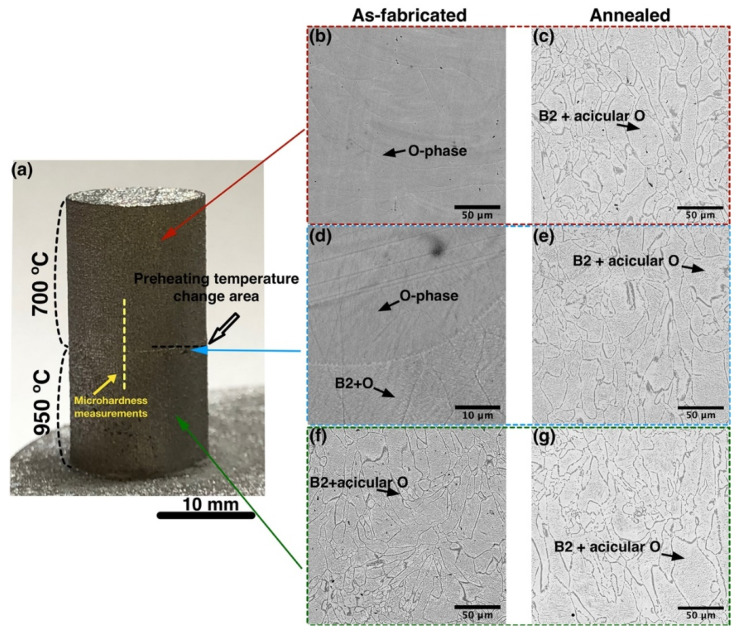
(**a**) A photograph of a microstructurally graded sample and BSE-SEM images showing microstructures of areas fabricated at (**b**,**c**) 700 °C, (**f**,**g**) 950 °C platform preheating, and (**d**,**e**) the transition area (**b**,**d**,**f**) before and (**c**,**e**,**g**) after annealing.

**Figure 4:**
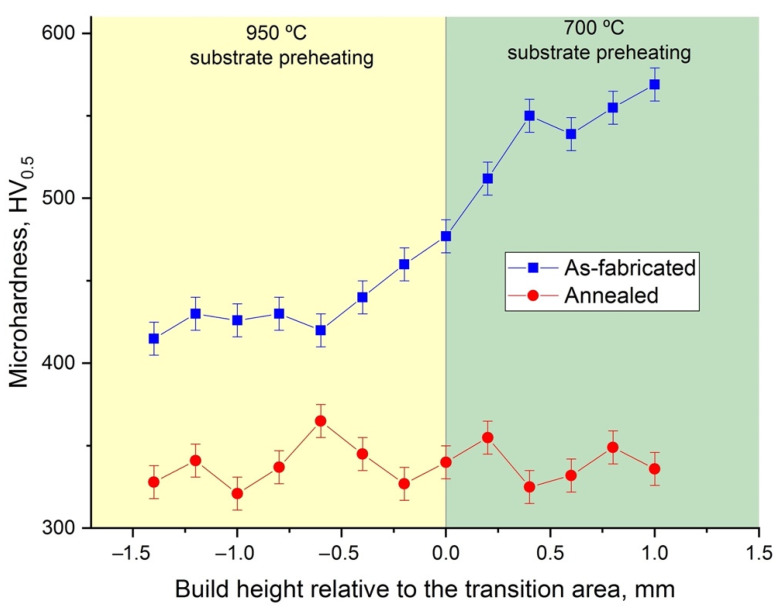
Microhardness distribution along the build direction for the microstructurally graded sample before and after annealing.

Using 950 °C and 700 °C platform preheating temperatures during the SLM process of the first and second halves of the sample, a microstructurally graded sample was fabricated as shown in Figure 3a. The bottom part had a B2 + O microstructure with fine acicular O-phase inside equiaxed prior B2 grains, as expected for 950 °C preheating temperature. The part of the sample fabricated at 700 °C preheating temperature had a fully-O microstructure. A transition zone could be found in the sample where the SLM process was paused and the preheating temperature changed from 950 °C to 700 °C. The fine B2 + O microstructure transformed into a fully-O microstructure as the height of the sample increased from 950 °C into the 700 °C preheating temperature zone. Microhardness measurements (Figure 4) along the build direction showed a steep increase in microhardness values along the transition zone, as the fully-O microstructure is characterized by higher hardness (about 560 HV_0.5_) compared with the B2 + O microstructure (about 430 HV_0.5_).

Annealing the sample with the graded microstructure at 1050 °C resulted in a homogeneous B2 + O microstructure (Figure 3c–g) with equiaxed B2 grains and fine acicular O-phase precipitates similar to the microstructure of the sample fabricated at 950 °C platform preheating. No significant differences in microstructures of the bottom, top, and transition areas of the sample were found, suggesting that heat treatment eliminated microstructural inhomogeneity of the alloy. At the same time, microhardness measurements of the annealed sample showed an even distribution of the values along the build direction. After heat treatment, the microhardness decreased to 340 ± 20 HV_0.5_ due to a decreased O-phase volume fraction.

#### 3.1.3. Effect of Hot Isostatic Pressing on Microstructure and Tensile Properties of the Orthorhombic Alloy Fabricated by SLM

**Figure 5:**
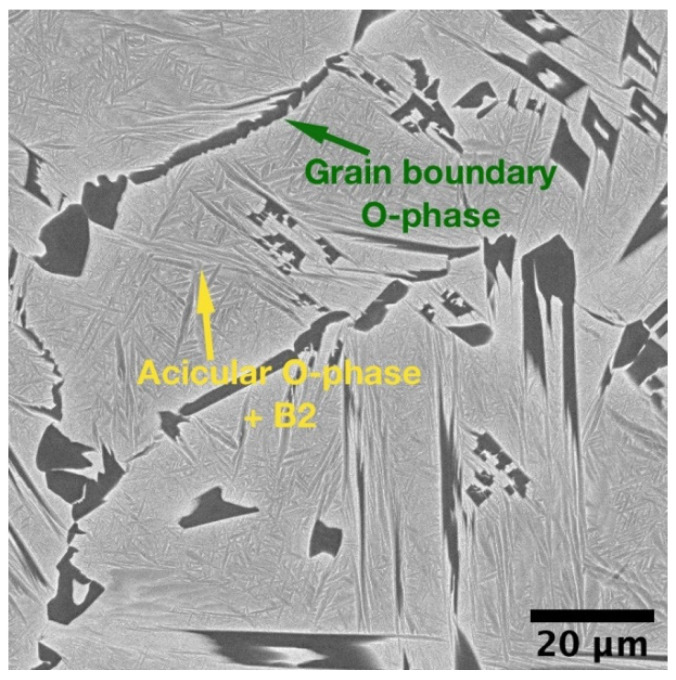
BSE-SEM image of the orthorhombic alloy obtained by SLM after HIP.

Figure 5 shows the microstructure of the orthorhombic alloy after HIP at 1050 °C. A B2 + O microstructure with equiaxed B2 grains and acicular O-phase was obtained, similar to the sample after annealing at the same temperature. At the same time, no residual defects in the form of micro-cracks were found in the images, suggesting that HIP allowed for the elimination the residual defects. As the result of B2 → O transformation and coarsening of O-phase precipitates during the HIP, the microhardness of the alloy decreased to 360 ± 10 HV HV_0.5_ compared with the as-fabricated condition. The HIPed alloy also exhibited lower microhardness compared with the annealed condition since the HIP temperature was higher and resulted in a coarser microstructure and higher volume fraction of the B2-phase, which has lower hardness compared with the O-phase [21].

The manufactured dog-bone tensile samples were machined and tested in as-fabricated and HIPed conditions. The results of tensile tests are shown in Table 2. In the as-fabricated condition, room temperature tensile strength (TS) reached 630 MPa while elongation at break (EL) could not be measured since the specimens demonstrated brittle fracture without reaching plastic deformation. Annealing of the specimens did not result in improved tensile behavior, and the annealed specimens also demonstrated brittle deformation at room temperature. Poor mechanical performance in the as-fabricated and annealed conditions can be attributed to the presence of defects such as microcracks leading to premature failure during the tensile tests.

**Figure 6:**
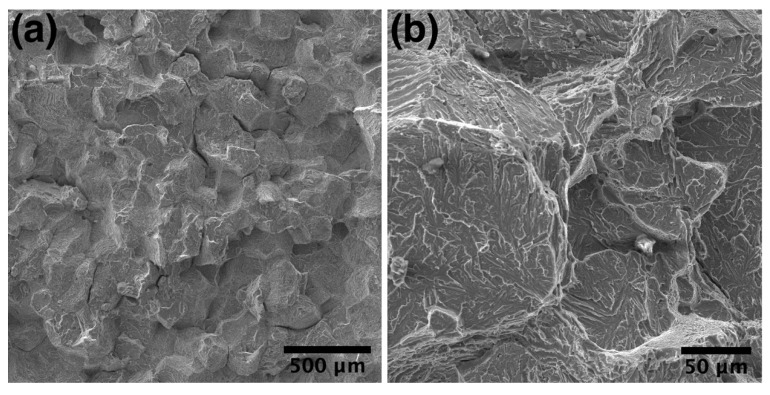
SEM-images of the fracture surface of the HIPed sample after RT tensile test: (**a**) general view, (**b**) higher magnification showing an intergranular fracture.

After HIP, the alloy demonstrated improved tensile performance both at room and elevated temperatures. Room temperature (RT) TS reached 1027 MPa, while TS reached 860 MPa and 770 MPa at 650 °C and 700 °C testing temperatures, correspondingly. The obtained values were close to the wrought or spark-plasma-sintered orthorhombic alloys. However, it should be noted that even after HIP, the obtained alloy demonstrated low room-temperature elongation, which might be attributed to the presence of the brittle O-phase along B2 grain boundaries. As can be seen in Figure 6, the fracture surface of the HIPed sample showed a brittle intergranular fracture, suggesting that the fracture was initiated at grain boundaries.

### 3.2. Microstructure and Microhardness of the Orthorhombic Alloy Produced with Multiple Laser Scanning

The scanning strategy with multiple laser exposures with each layer or every third layer additionally scanned for 10 times was utilized to evaluate the feasibility of microstructure modification by laser-induced heat treatment. Figure 6 shows the microstructure of the samples obtained with a single laser exposure, multiple laser exposures for each layer, and every 3rd layer at 700 °C platform preheating, and the samples produced with a single laser exposure at 800 °C and 950 °C platform preheating.

**Figure 7:**
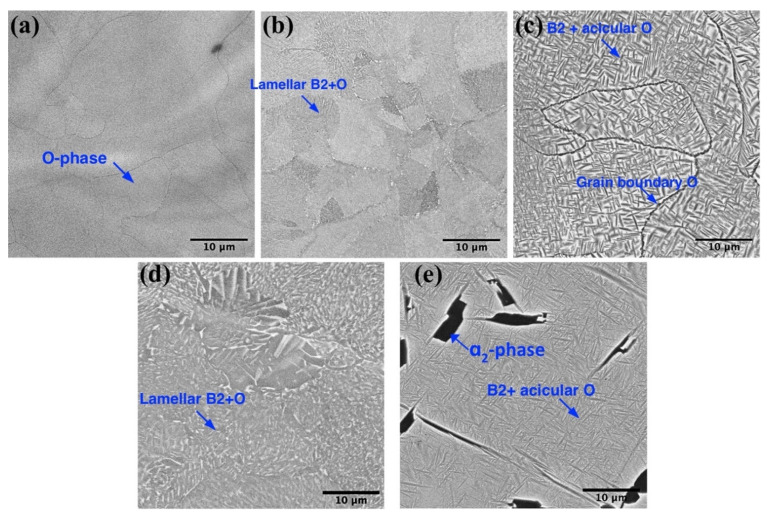
BSE-SEM-images showing microstructure of the samples: (**a**) 700-x0, (**b**) 700-x10-3, (**c**) 700-x10, (**d**) 800-x0, (**e**) 950-x0.

**Figure 8:**
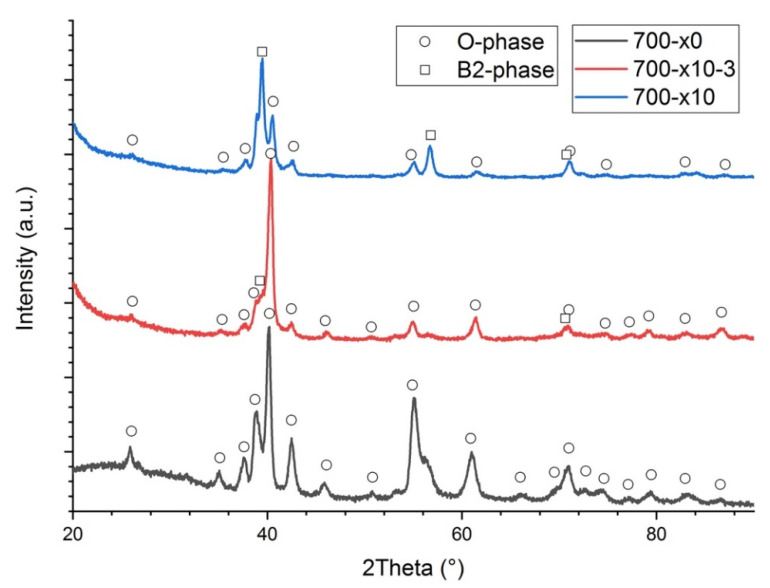
XRD results for the samples 700-x0, 700-x10-3, 700-x10.

Applying additional laser scanning induced in situ annealing during the SLM process and promoted O-phase decomposition and formation of the B2 + O microstructure. When every 3rd layer was additionally scanned 10 times, a fine lamellar microstructure consisting of B2 + O lamellas within prior O grains was obtained (Figure 7b). This microstructure demonstrated decreased microhardness (Table 3) compared with the fully-O sample obtained at 700 °C and single-laser scanning (Figure 7a) due to the presence of the B2 phase. The decomposition of the O-phase into B2 and O phases induced by multiple-laser exposure was also confirmed by the XRD results, as can be seen in Figure 8. When each layer was additionally scanned during the SLM process, B2 phase volume fraction significantly increased and the O phase transformed into acicular-shaped precipitates, suggesting that additional laser scanning induced heating above O ⟷ B2 phase transformation temperature. An increased B2 phase volume fraction and coarser O phase precipitates resulted in reduced microhardness values. In general, applying additional laser scanning promoted in situ annealing of the orthorhombic alloy during the SLM process and allowed for the obtainment of a microstructure similar to the alloy produced at higher platform preheating temperatures.

## 4. Conclusions

In this work, microstructure and mechanical properties of titanium orthorhombic alloy fabricated by SLM with high-temperature substrate preheating were investigated. The microstructural homogeneity of the alloy along the build direction was evaluated. It was shown that when high-temperature substrate preheating is used in the SLM process, the uneven temperature distribution might lead to inhomogeneous microstructures and the formation of micro defects.

The feasibility of mitigating the microstructural inhomogeneity was investigated by fabricating Ti2AlNb-based alloy samples with the graded microstructure and subjecting them to annealing and HIP. Annealing allowed us to achieve a homogeneous B2 + O microstructure of the alloy, while HIP drastically improved tensile properties, resulting in tensile strength of 1027 MPa, 860 MPa, and 770 MPa at room temperature, 650 °C, and 700 °C, correspondingly.

The scanning strategy with multiple laser exposures was utilized to promote in situ annealing of the alloy. Scanning each layer ten additional times transformed fully-O microstructures into B2 + O microstructures, showing that applying additional laser scanning can be used to tailor the microstructure of the alloy.

## Figures and Tables

**Table 1:** SLM process parameters used to produce the samples with multiple-laser scanning.

Sample	Main Scan				Additional Scans				Preheating Temperature, °C
	P, W	S, mm/s	HD, mm	L, mm	P, W	S, mm/s	HD, mm	Number of Scans	
700-x0	140	850	0.12	0.03	140	2000	0.35	0	700
800-x0								0	800
950-x0								0	950
700-x10								10 (every layer)	700
700-x10-3								10 (every 3rd layer)	700

**Table 2:** Comparison of mechanical properties of titanium orthorhombic alloys obtained by different techniques.

Fabrication Technique	TS (RT), MPa	TS (650 °C), MPa	TS (700 °C), MPa	EL (RT), %	EL (650 °C), %	EL (700 °C), %
SLM, 950 °C preheating, as-fabricated (this work)	630 ± 30	n/e	n/e	–	n/e	n/e
SLM, 950 °C preheating, annealed (this work)	690 ± 20	n/e	n/e	–	n/e	n/e
SLM, 950 °C preheating, HIPed (this work)	1027 ± 50	860 ± 30	770 ± 30	1.1 ± 0.1	5.8 ± 0.5	6.0 ± 0.5
Vacuum hot pressing sintering [22]	628–869	429–670	n/e	2.5–3.9	4.0–8.0	n/e
Spark plasma sintering [23]	1105	797	n/e	9.4	12.8	n/e
Wrought and annealed [24]	1110	n/e	850	9.0	n/e	5.5

**Table 3:** Microhardness of the samples fabricated using single and multiple-laser scanning strategies at different platform preheating temperatures.

Sample	700-x0	700-x10	700-x10-3	800-x0	950-x0
Microhardness, HV_0.5_	525 ± 15	348 ± 10	456 ± 15	449 ± 15	435 ± 15

## Data Availability

The data presented in this study are available on request from the corresponding author.
